# Effects  of rural–urban residence and education on intimate partner violence among women in Sub-Saharan Africa: a meta-analysis of health survey data

**DOI:** 10.1186/s12905-021-01286-5

**Published:** 2021-04-13

**Authors:** Maria Sarah Nabaggala, Tarylee Reddy, Samuel Manda

**Affiliations:** 1 Infectious Diseases Institute, College of Health Sciences, Makerere University, Kampala, Uganda; 2 Biostatistics Research Unit, South African Medical Research Council, Durban, South Africa; 3 School of Mathematics, Statistics and Computer Science, University of KwaZulu-Natal, Pietermaritzburg, 3201 South Africa; 4 Department of Statistics, University of Pretoria, Pretoria, South Africa

**Keywords:** Intimate partner violence, Sub-Saharan Africa, Meta-analysis, Residence differences, Education level difference

## Abstract

**Background:**

Intimate Partner Violence (IPV) against women is a major public health and human rights problem worldwide. Sub-Saharan Africa (SSA) has one of the highest prevalence of IPV against women in the world. This study used meta-analysis to obtain pooled rural–urban and education attainment differences in the prevalence of IPV among ever-partnered women in SSA, and assessed whether the differences in IPV depended on the SSA region or period or women’s age.

**Methods:**

We analysed IPV data on 233,585 ever-partnered women aged 15–49 years from 44 demographic and health surveys conducted between 2000 and 2018 in 29 SSA countries. Random-effects meta-analyses were used to estimate overall rural–urban residence and educational differences in IPV rates among the women in SSA. Subgroup analyses were also done to investigate the sources of heterogeneity in the overall meta-analysis findings.

**Results:**

The pooled prevalence of intimate partner violence was estimated to be 41.3% (37.4–45.2%). Regionally, the highest prevalence of IPV was in Middle Africa (49. 3%; 40.32–58.45), followed by East Africa (44.13%; 36.62–51.67), Southern Africa (39.36%; 34.23–44.49), and West Africa (34.30%; 27.38–41.22). The risks of experiencing IPV were significantly higher if the women had less than secondary education (RR = 1.12; 95% CI  1.07–1.22) compared to those with at least a secondary education. Generally, women who resided in a rural area had their risks of experiencing IPV increased (RR = 1.02; CI 0.96–1.06) compared to those who resided in urban areas, but the IPV increases were only significant in East Africa (RR = 1.13; CI 1.07–1.22).

**Conclusion:**

In sub-Saharan Africa, intimate partner violence against women is widespread, but the levels are much higher among women with lower levels of education and residing in rural areas. Our findings have provided additional support to policies aimed at achieving SDG goals on the elimination of all forms of violence against women and girls in sub-Saharan Africa. For example, policies that advocate improved educational attainment, especially among women and communities in rural areas.

**Supplementary Information:**

The online version contains supplementary material available at 10.1186/s12905-021-01286-5.

## Background

Intimate Partner Violence (IPV) is violence that occurs between people in intimate relationships and includes physical, sexual, and emotional violence. Women who are exposed and experience IPV suffer from higher levels of poor physical and mental health, poor psychological health, depression, anxiety, and phobias, and are more likely to harbor thoughts of suicide and attempted suicide [1–4]. Also, IPV among women can result in adverse sexual and reproductive health problems for women including exposure to sexually transmitted infections such as HIV, negative pregnancy outcomes, unintended and/or unwanted pregnancies [4–9]. There is now a global recognition that violence against women is a significant public health concern and a violation of women’s human rights [1, 2].

Women’s experience of IPV is widespread throughout much of sub-Saharan Africa (SSA) [1]. At 36.6%, the WHO African Region (mostly SSA countries) is one of the three WHO regions with the highest prevalence of physical or sexual intimate partner violence among the ever-partnered women. The other two regions being WHO South-East Asia (37.7%) and Eastern Mediterranean (37.0%). These three WHO regions account for 37% of all ever-partnered women who experience intimate partner violence, far exceeding the global average (30%) [2]. Even within the WHO African Region, IPV against women varies between and within countries, and between SSA regions [2, 3, 10]. Countrywide, lower levels of IPV against women are generally found in Comoros, Burkina Faso, and the Gambia while higher levels of IPV are experienced by women in Cameroon, Uganda, and Congo DR. Regionally, East Africa bears the highest burden of IPV against women.

In the context of sub-Saharan Africa, few country-level studies have been carried out looking at how residence and education are associated with IPV [11–15]. These studies have shown that increased women’s levels of education are associated with a reduction in their risk of IPV. Also, male partners who have higher levels of education tend to be significantly associated with a lower likelihood of IPV perpetration [16, 17]. The rural–urban residence has also been found to be associated with IPV, with rural women experiencing  higher rates of IPV when compared to urban residence [14, 18–21]. These findings have been linked to the fact that the highest percentage (63%) of the African population live in remote rural areas [18], further away from available resources and influence of the rule of law prohibiting gender-based violence, which make enforcement of strict laws against violence very limited. Also, inflexible norms that condone violence in families change at a slower rate in rural areas compared to urban areas [19, 20], This results in rural women being more prone to IPV compared to their urban counterparts.

Other risk factors for IPV in Africa include alcohol consumption, history of child abuse, and socioeconomic conditions such as unemployment [1, 20, 22–24]. Particularly, for alcohol consumption, men with a predisposition for excessive alcohol or substance abuse are more likely to inflict violence onto their partners [15, 20, 25–29]. Also, childhood history of abuse [25], through exposure to parental violence, is associated with spouse abuse in both men and women [22, 25, 26]. Limited economic or social empowerment may render women vulnerable to being unnecessarily obedient to their spouses [27, 31]. The likelihood of all forms of IPV decreases with women playing a more active role in household decision-making [32]. Given that the SSA is the poorest in the world, with up to 80% of the population living under the global poverty threshold [18], it is unsurprising that the high prevalence of IPV in the region is linked to the economic vulnerability of women [19]. Patriarchal beliefs relating to gender roles are very prevalent in many regions of Africa [10, 20], and these beliefs promote men’s rights to exercise power and control over women in sexual relationships, especially marriage [14, 33]. Attitudes toward partner violence are highly associated with cultural beliefs [15, 34–36].

Data on IPV from nationally representative population-based surveys, mainly the Demographic and Health Surveys (DHS), have been used in sub-Saharan Africa for national and multi-country comparisons of IPV [4–9, 26, 37–39]. The findings from these studies present a mixed association of rurality, lower levels of education, and IPV against women in the SSA region. Hence, we have undertaken this meta-analysis to determine the association between rural–urban residence, educational attainment, and IPV against women in the region. To the best of our knowledge, there has not been a study that summarized urban–rural and educational level IPV differences using DHS datasets in the sub-Saharan African region. By conducting a meta-analysis of the prevalence of IPV, which is a very important health and human rights issue, the study will provide greater objectivity, generalizability, and increased power for the prevalence of IPV by including all the available evidence from nationally representative data. The findings will provide evidence regarding groups of women that are most affected by IPV. This in turn will support effective interventions on IPV in line with the 2030 Agenda for Sustainable Development Goals (SDG), especially SDG 5, which addresses gender equality and the empowerment of women and girls.

## Methods

### Data

The study used IPV data reported by 233,385 ever-married women aged 15–49 in 44 Demographic and Health  (DHS) conducted between 2000 and 2018 across 29 countries in the sub-Saharan African region. These surveys  are nationally representative household surveys that provide data for a wide range of population, health, and nutrition indicators in low- and middle-income (LMICs). The Demographic and Health Surveys are implemented by the Demographic Health Survey Program, which is funded by the United States Agency for International Development (USAID). Details of DHS sampling design and data accessibility are found at www.measuredhs.org. The DHS surveys generally have high national response rates and use uniform high and quality interviewer training and data collection instruments across countries and over time which allow for detailed national and sub-national population comparisons of health status [40]. Briefly, most of the surveys employed stratified two-stage cluster sampling to accrue the required number of  women. The countries included in this study were chosen based on the availability of data on the domestic violence module, which has a set of standard questions, thus allowing for comparison between countries [41].

### Measures of IPV

Domestic violence data are collected using standardized modules, in every second or third of the sampled households. Following the World Health Organization’s guidelines on the ethical collection of information on domestic violence, only one eligible woman per household is randomly selected for the module, and the module is not implemented if privacy could not be assured. For the IPV data, measures for emotional, physical, and sexual forms of IPV were collected using the modified version of the Conflict Tactic Scale (CTS) [42]. The modified CTS is comprised of 13–16 items, each with a “Yes” or “No” response. The items are grouped into Emotional, Physical, and Sexual Violence domains [5, 43]. A domain was categorized as ever experienced the respective violence, coded “1 = Yes” if any item constituting that domain had a response in the affirmative, otherwise it was coded “0 = No”. The main outcome variable, namely the overall IPV was categorized as “1 = Yes” if any of the three domains was coded “1 = Yes”, otherwise it was coded “0 = No.”

We quantified IPV differences by the rural–urban residence and education categorised as low education (Primary or below) and high education (Secondary or above). The number of women interviewed for IPV and the number who had experienced IPV were extracted from survey data to determine rural–urban and low education-high education relative risks (RRs) of IPV among women. The analyses were further stratified by four regions of sub-Saharan Africa, namely: Eastern Africa, Western Africa, Southern Africa, and Middle Africa; the period in which the study was conducted (2000–2009 and 2010–2018) and women’s age group (15–24 and 25–49 years).

### Statistical analysis

Random effects meta-analyses were implemented to produce overall estimates of the prevalence of IPV against women and its relation to geographic type and literacy. Results are presented using forest plots showing pooled prevalence and association in each region and period and their associated 95% confidence intervals (CI’s) for each study. Heterogeneity between reported prevalence rates was assessed by conducting the Chi-square test, Q-statistics, and I^2^ test [44]. Based on the results of the statistical test, if significant heterogeneity is observed among the included studies then a random-effects meta-analysis model would be conducted to estimate overall pooled effects for the whole SSA and within each of the four regions. The reference category for residence type was rural and the reference category of at most primary education for education was used when estimating relative risks. Results are presented using forest plots that show point prevalence and relative risk estimates as well as 95% confidence intervals for each survey dataset and pooled results. To investigate the sources of heterogeneity in the meta-analysis findings, subgroup meta-analyses were performed by the Africa region, period, and women’s age group. All analysis was conducted using Stata 14.0 using the *metan* command.

## Results

A total of 233,585 ever-partnered women aged 15 to 45 years from the 44 DHS surveys across 29 countries in the sub-Saharan Africa region provided data on their experience of IPV. Of these women, 74,956 were residing in urban areas, and 64,200 had attained at least a secondary education. The weighted prevalence, with associated 95% confidence intervals, of any form of IPV experienced by the interviewed women, are shown in Table 1. Also shown are the total number of the women interviewed on IPV as well as IPV experience disaggregated by educational attainment and rural–urban residence.

**Table 1:** Prevalence and associated 95% confidence interval (CI) of any intimate partner violence by women educational attainment or rural–urban residence as well as the number of women interviewed for intimate partner violence and DHS survey

Country and year	None to primary education		Secondary or higher education		Urban residence		Rural residence		National		IPV sample	DHS sample (15–49 years)
	IPV	95% CI	IPV	95% CI	IPV	95% CI	IPV	95% CI	IPV	95% CI		
Angola 2015	40.0	38.2–41.7	40.5	38.1–43.0	42.1	40.2–43.9	37.6	35.2–40.0	40.1	38.6–41.6	7669	14,379
Burkina Faso 2010	15.5	14.6–16.4	18.6	15.9–21.7	18.2	16.6–19.9	14.8	13.7–15.9	15.7	14.8–16.6	10,001	17,087
Burundi 2016	52.5	51.0–54.0	31.1	28.1–34.3	40.7	37.7–43.6	51.8	50.2–53.4	50.0	48.6–51.3	7366	17,269
Cameroon 2004	48.8	44.5–53.1	55.5	51.3–59.7	52.2	48.5–55.9	50.0	44.1–55.9	51.0	47.1–54.8	2572	10,656
Cameroon 2011	55.3	53.1–57.5	61.1	58.4–63.7	57.5	55.1–59.9	57.3	54.9–59.8	57.4	55.7–59.1	4001	15,426
Chad 2014	29.5	27.7–31.3	33.9	29.2–39.0	31.4	27.7–35.3	29.4	27.5–31.4	29.9	28.2–31.6	3807	17,719
Comoros 2012	11.6	9.9–13.5	9.3	7.6–11.3	12.2	10.0–14.7	9.8	8.1–11.8	10.8	9.4–12.3	2527	5329
Congo D R 2007	67.1	64.6–69.6	68.2	65.0–71.2	69.2	66.4–71.8	66.4	63.5–69.2	67.5	65.4–69.5	2850	9995
Congo D R 2013	56.7	54.5–58.9	55.5	52.9–58.1	58.6	55.7–61.4	55.4	53.1–57.7	56.3	54.5–58.1	5684	18,827
Cote dvoire 2011	31.2	29.7–32.8	30.5	26.7–34.6	32.9	30.7–35.2	30.1	28.3–32.1	31.2	29.7–32.7	5006	10,060
Ethiopia 2016	30.9	29.1–32.7	25.7	22.4–29.4	27.7	24.9–30.6	31.0	29.1–33.0	30.1	28.6–31.8	4720	15,683
Gabon 2012	57.3	54.9–59.7	55.7	53.3–58.0	54.6	52.2–57.0	59.4	56.8–62.0	56.4	54.6–58.2	4130	8422
Gambia 2013	29.4	27.3–31.5	28.0	24.9–31.4	28.1	25.5–30.9	29.7	27.3–32.3	29.1	27.3–30.9	3535	10,233
Ghana 2008	39.0	36.0–42.2	38.6	35.2–42.1	41.2	37.5–45.0	37.3	34.4–40.3	38.8	36.5–41.2	1831	4916
Kenya 2003	47.4	45.2–49.5	41.8	38.7–44.9	41.8	38.7–45.0	47.7	45.4–49.9	45.9	44.1–47.8	4310	8195
Kenya 2008	48.4	46.3–50.6	34.3	31.5–37.1	37.9	34.9–41.0	47.3	45.0–49.6	44.6	42.8–46.5	4902	8444
Kenya 2014	46.9	45.1–48.6	39.6	36.9–42.4	42.7	40.2–45.2	46.0	44.2–47.9	44.8	43.3–46.3	4512	31,079
Liberia 2007	48.7	46.1–51.2	50.8	45.9–55.6	53.9	50.0–57.7	46.4	43.4–49.5	49.1	46.7–51.5	3909	7092
Malawi 2004	30.6	29.3–31.9	27.0	24.1–30.2	29.3	26.2–32.5	30.4	29.1–31.8	30.3	29.1–31.5	8291	11,698
Malawi 2010	38.2	36.6–39.8	36.3	32.9–39.7	39.5	35.4–43.8	37.7	36.1–39.3	37.9	36.4–39.4	5369	23,020
Malawi 2015	43.4	41.7–45.1	34.5	31.9–37.3	40.8	37.6–44.1	41.6	39.9–43.3	41.5	40.0–42.9	5406	24,562
Mali 2012	45.1	42.6–47.7	42.6	36.8–48.6	44.1	39.7–48.5	45.1	42.2–48.1	44.8	42.4–47.3	3120	10,424
Mozambique 2011	45.9	44.3–47.5	48.2	44.7–51.7	51.4	49.1–53.7	43.6	41.6–45.5	46.2	44.7–47.7	5824	13,745
Mozambique 2015	23.0	21.2–24.9	32.0	28.2–36.2	31.7	28.7–34.9	20.4	18.4–22.4	24.7	23.0–26.4	2874	6946
Namibia 2013	39.7	35.0–44.6	31.8	28.7–34.9	34.1	30.3–38.1	34.6	31.0–38.5	34.3	31.7–37.1	1447	9176
Nigeria 2008	29.6	26.0–33.5	29.7	26.3–33.5	26.8	23.2–30.6	30.9	26.9–35.2	29.7	26.6–33.0	19,204	33,385
Nigeria 2013	23.8	22.4–25.2	29.5	28.0–31.1	27.3	25.5–29.2	25.1	23.6–26.7	25.9	24.7–27.1	22,253	38,948
Rwanda 2005	39.7	37.6–41.7	27.8	22.2–34.3	37.7	33.2–42.3	38.9	36.7–41.0	38.7	36.7–40.6	2537	11,321
Rwanda 2014	42.2	39.7–44.7	25.1	20.1–30.9	34.0	29.5–38.9	41.8	39.2–44.5	40.2	37.9–42.6	1905	13,497
Sao Tome 2008	37.0	33.7–40.5	31.8	27.4–36.6	36.9	31.7–42.5	35.1	31.6–38.8	35.9	32.9–39.0	1728	2615
Senegal 2017	28.2	26.2–30.2	20.2	16.4–24.5	26.3	23.7–29.0	27.5	25.2–29.8	27.0	25.3–28.8	2660	16,787
Sierra leone 2013	48.1	46.1–50.2	52.1	48.1–56.1	49.6	46.4–52.8	48.3	46.0–50.6	48.7	46.9–50.6	4307	16,658
South Africa 2016	30.1	26.3–34.1	22.8	21.3–24.3	23.1	21.2–25.1	24.6	22.5–26.8	23.7	22.3–25.2	4003	8514
Tanzania 2010	45.6	43.8–47.4	21.5	18.5–24.8	39.6	36.4-43.0	43.5	41.6-45.6	42.7	41.0-44.4	5687	10139
Tanzania 2015	48.5	47.0–50.1	31.2	28.6–34.0	45.2	42.7–47.8	45.6	44.0–47.3	45.5	44.1–46.9	7597	13266
Togo 2013	39.5	37.5–41.4	30.8	28.1–33.7	31.5	28.9–34.1	40.7	38.5–43.0	37.5	35.8–39.3	5374	9480
Uganda 2006	68.4	64.3–72.2	54.1	47.3–60.7	56.6	49.4–63.6	67.8	63.9–71.6	66.2	62.9–69.4	1748	8531
Uganda 2011	61.1	58.3–63.8	46.7	41.8–51.6	52.4	47.7–57.0	59.5	56.6–62.4	57.8	55.2–60.2	1702	8674
Uganda 2016	60.7	59.3–62.1	45.3	43.0–47.6	47.3	44.3–50.3	59.4	58.0–60.8	56.9	55.6–58.2	7536	18506
Zambia 2007	52.9	49.1–56.6	53.9	49.3–58.4	58.1	53.8–62.2	50.4	46.8–53.9	53.1	49.8–56.4	4229	7146
Zambia 2013	49.4	47.9–50.8	44.7	42.8–46.6	48.7	46.9–50.5	47.3	45.6–48.9	47.8	46.6–49.1	9403	16411
Zimbabwe 2005	47.9	45.5–50.3	45.3	43.1–47.6	39.5	36.6–42.6	49.5	47.4–51.5	46.5	44.8–48.2	4962	8907
Zimbabwe 2010	46.1	43.6–48.6	39.9	38.1–41.7	39.3	36.7–42.0	43.5	41.7–45.4	42.2	40.7–43.7	5280	9171
Zimbabwe 2015	46.7	44.2–49.2	43.3	41.6–45.0	44.3	42.1–46.5	44.3	42.4–46.2	44.3	42.9–45.8	5800	9955

**Fig. 1:**
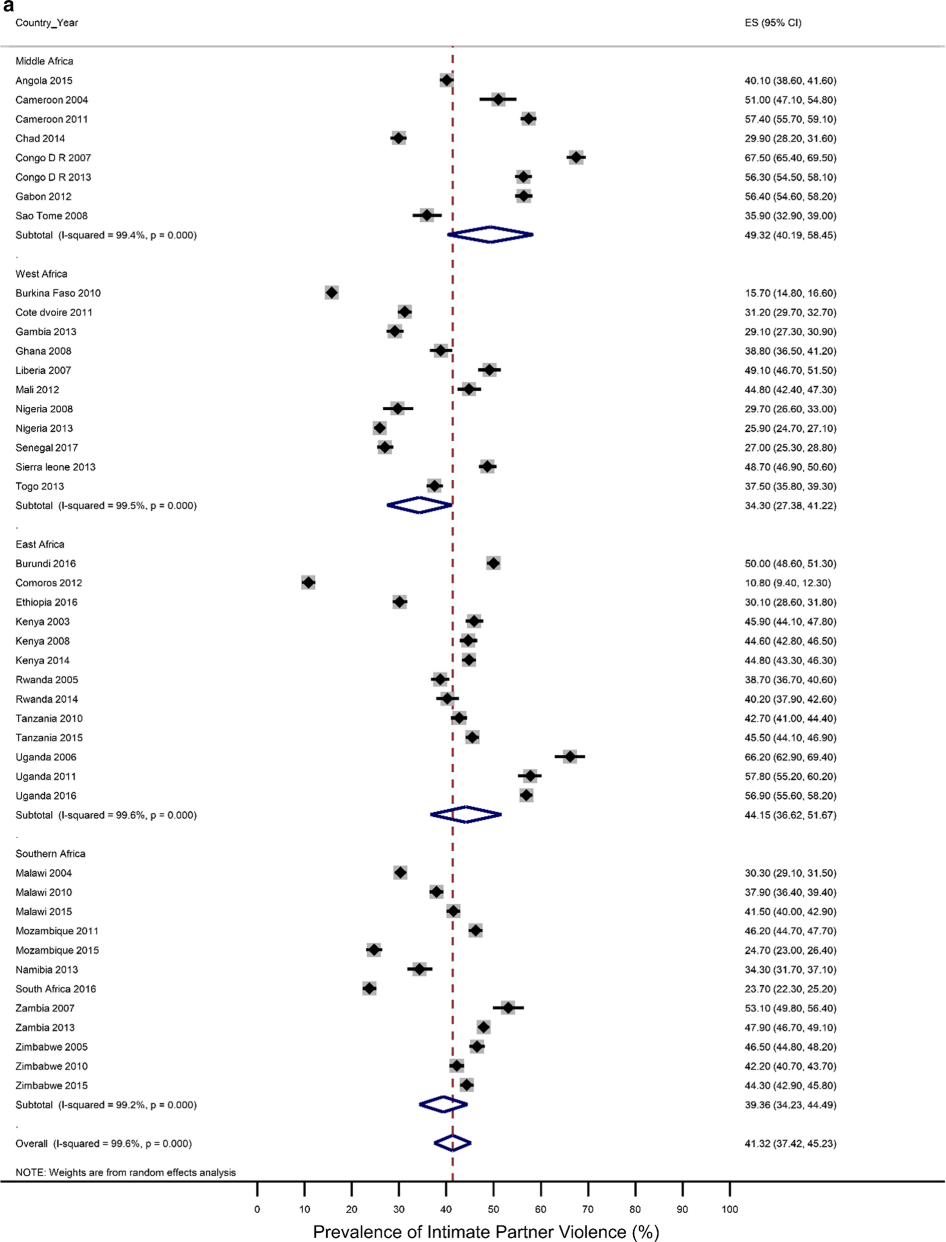

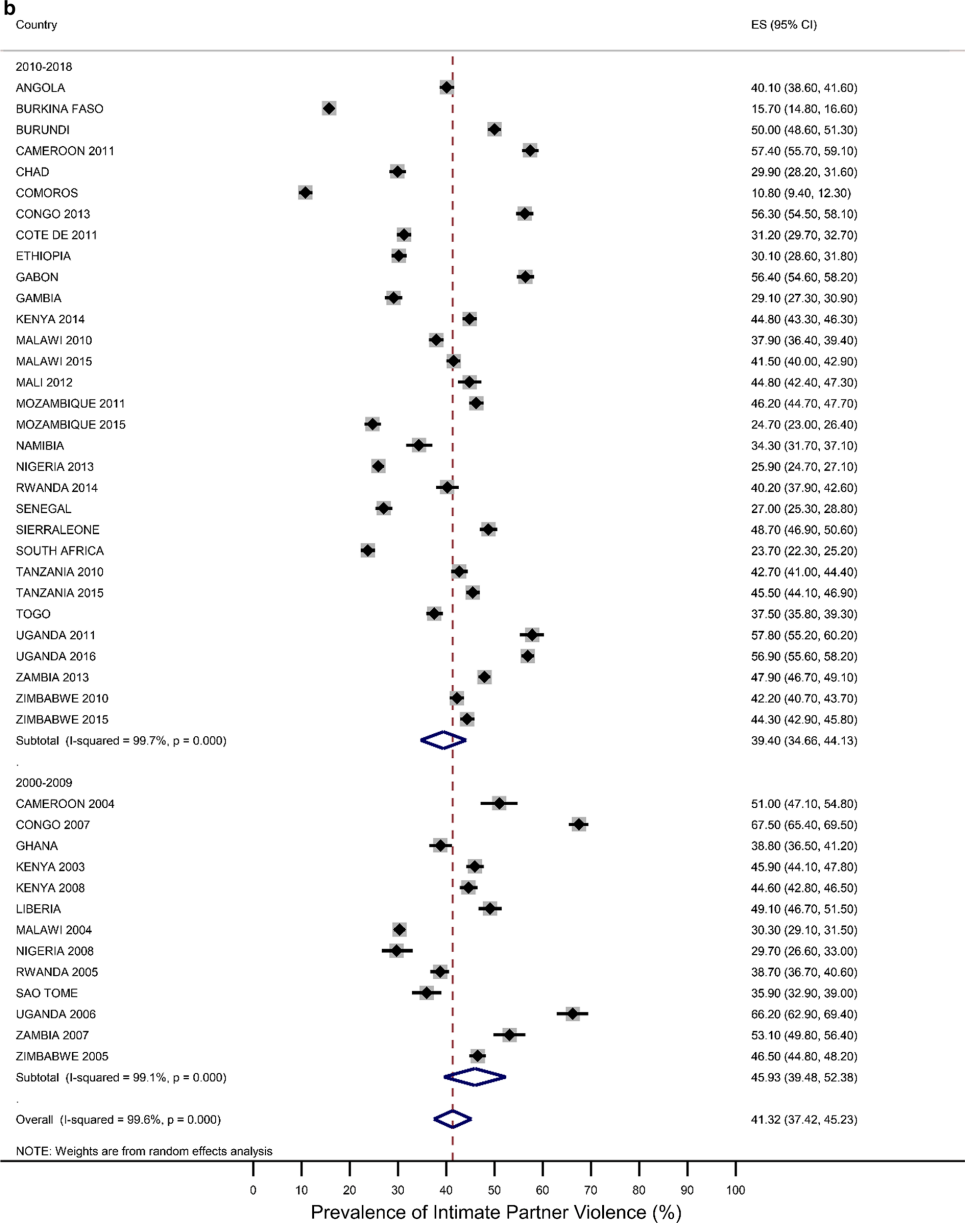
**a** Prevalence of any Intimate Partner Violence according to the region of sub-Saharan Africa. The dotted vertical line represents the pooled prevalence, with its associated 95% confidence interval. **b** Prevalence of any Intimate Partner Violence according to period. The dotted vertical line represents the pooled prevalence, with its associated 95% confidence interval

Five countries (Kenya, Malawi, Tanzania, Uganda, and Zimbabwe) had three surveys conducted in the study period; six had two surveys (Cameroon, Congo Democratic Republic, Mozambique, Nigeria, Tanzania, Zambia), while the rest had one survey. Most surveys (n = 32) were conducted between 2010 and 2018, with the remaining surveys conducted from 2000 to 2009. Survey-specific and pooled prevalence estimates of IPV against women are presented in Figs. 1a, b by the region of SSA and period, respectively. The included studies exhibited significant heterogeneity (Q statistic = 6557, I2 = 99.6, p < 0.001).

Figures 1a, b show that there was a large variation in the national prevalence of any IPV, ranging from 10.8% in Comoros and 15.7% in Burkina Faso to 66.2% in Uganda and 67.5% in the Democratic Republic of Congo. Mean and median country-level IPV prevalence was 42.45% and 41.3%, respectively. The IPV prevalence was negatively associated with increasing years (results not shown). The pooled prevalence of experiencing partner violence among the ever-married women was estimated at 41.3% [95% CI 37.4–45.2), with emotional violence 27.4% (23.6–30.2) and physical violence 31.5% (27.9–35.1)] being the most common types, followed by sexual violence (12.9%;11.0–14.8). The sub-region results showed that the highest pooled estimates of IPV were in Middle Africa [49. 3%; 40.32–58.45)], followed by East Africa (44.13%; 36.62–51.67), Southern Africa (39.36%; 34.23–44.49) and West Africa (34.30%; 27.38–41.22. Regional inequalities were observed regarding emotional and physical violence. For example, physical violence was highest in the Middle African region (39.77%; 32.85–46.70) while it was lowest in the Western region (23.15%;27.4–18.50–27.71) (Results not shown). With regards to sexual violence, the Eastern region (16.6%; 12.09–20.51) and the Middle region (16.43%; 12.04–20.83) had similar rates, which were higher compared to the rates in the Southern region (12.32%; 9.01–15.64) and the Western region (6.3%;4.6–7.9) (Results not shown). The period analysis showed that the overall IPV prevalence was lower in the later period of 2010–2018 (39.4%; 34.7–44.1) than in the earlier period, 2000–2009 (45.9%; 39.5–52.4).

### Rural–urban residence and educational attainment differences in IPV

**Fig. 2:**
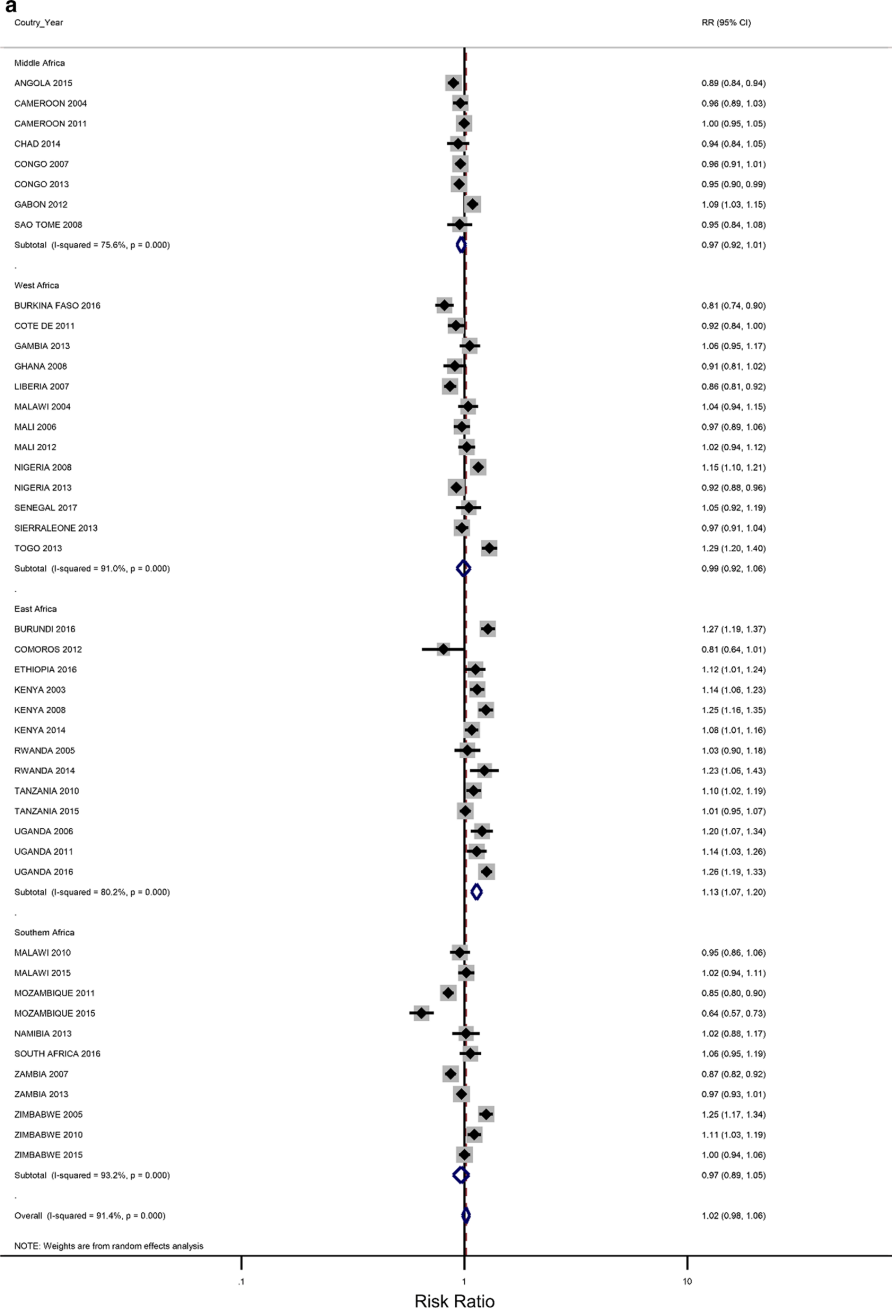

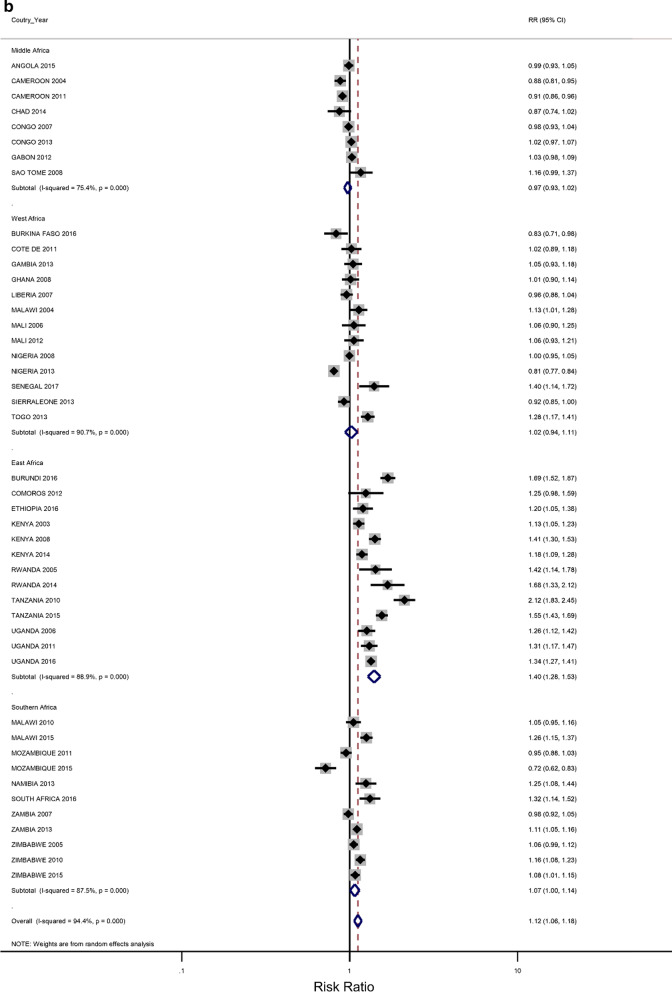
**a** The excess of Intimate Partner Violence prevalence among women in rural areas, compared to women in urban areas, by region of Africa. The dotted vertical line represents the pooled risk ratio, with its 95% confidence interval. The solid vertical line at the value of 1 represents no difference in IPV rates between the types of residence. **b** The excess of Intimate Partner Violence prevalence among women with less than a secondary education, compared to women with at least a secondary school education, by region of Africa. The dotted vertical line represents the pooled risk ratio, with its 95% confidence interval. The solid vertical line at the value of 1 represents no difference in IPV rates between the education levels

The prevalence of any IPV was generally higher among women living in rural areas (60% of the surveys) and the less-educated women (73% of the surveys) (Additional file 1: Figures A1–A2). The corresponding relative risks (RRs) for the prevalence of IPV among rural women compared to urban women, and among the least educated compared to the highly educated are shown in Fig. 2a, b). In the pooled analysis, IPV prevalence amongst women in rural communities was higher than for women in urban communities (RR = 1.02 and 95% confidence interval (CI) 0.96–1.06), but the association was only significant in the Eastern Africa region (RR = 1.13; 1.07–1.22). The pooled IPV among the less educated women was significantly greater than that of  women with at least a secondary education (RR = 1.12; 107–1.22) and the same was noted in the Eastern and Southern African regions (1.40; 1.28–1.5 and 1.07; 1.00–1.14, respectively).

### Stratified and Sensitivity analyses

In our analyses, the results have been presented for the overall age group 15–49 years that were eligible for standard demographic and health surveys. We attempted to minimize the influence of the region (of African) and period on the association between level of education and IPV, and the rural–urban residence and IPV using stratified meta-analyses. The estimated associations could also be confounded by age, a known cofounder for IPV against women [45, 46]. The age distribution was found to be significantly different between the countries, years, and regions of Africa. Thus, we performed a meta-analysis within each of the two groups: 15–24 and 25–49 years. For each age group, study-specific and pooled prevalence estimates of IPV against women are presented in Additional file 2: Figures B1–B4 by the region of SSA and period. The prevalence of the overall pooled IPV among 15–24 and 25–49-year-old women was 38.9% (34.97–42.94) and 41.6% (37.73–45.38), respectively. The sub-region analysis showed that young women residing in the Middle African region experienced the highest levels of IPV (48.7%; 39.3–58.0) while the lowest prevalence was observed in the Western region (31.0%; 25.5–36.6). Further sub-region analysis resulted in a similar regional pattern for the older women with an estimated IPV of 49.7% (41.2–58.1) and 33.9% (28.6–39.2), respectively.

**Fig. 3:**
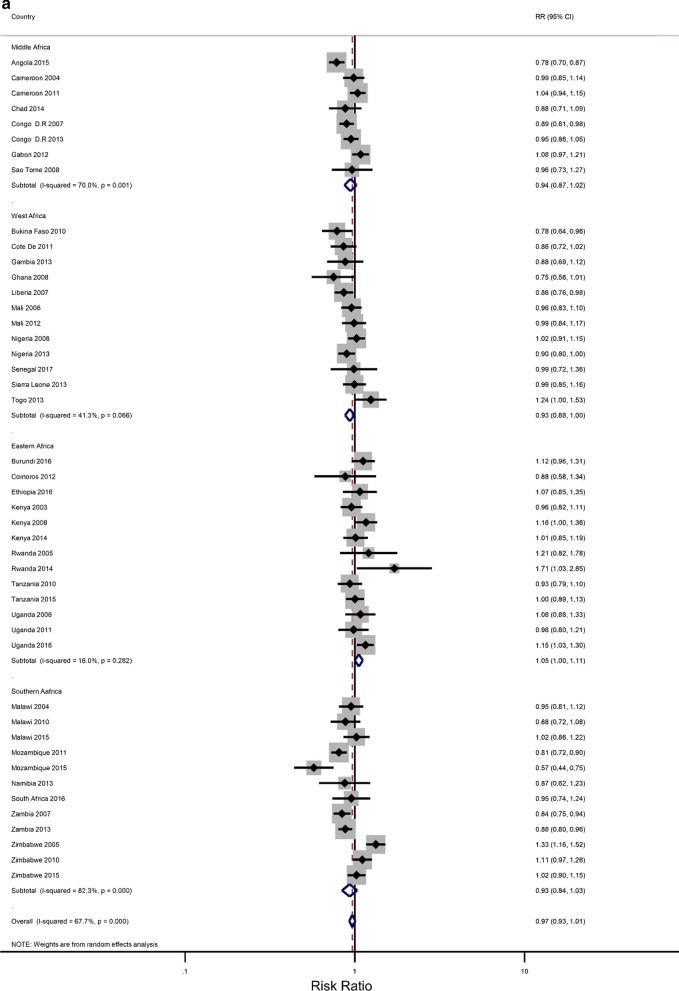

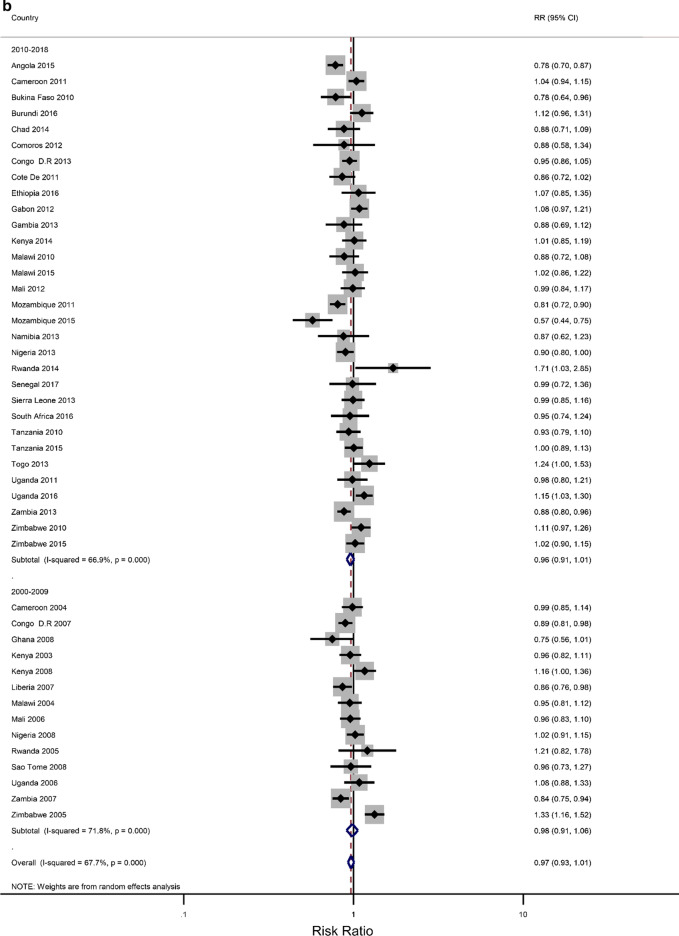

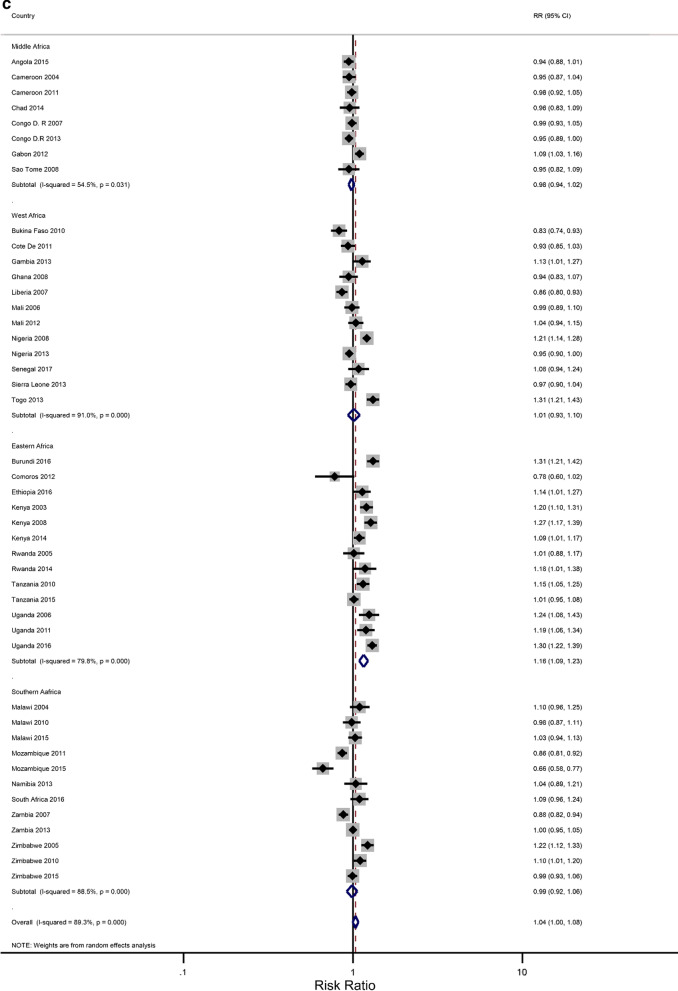

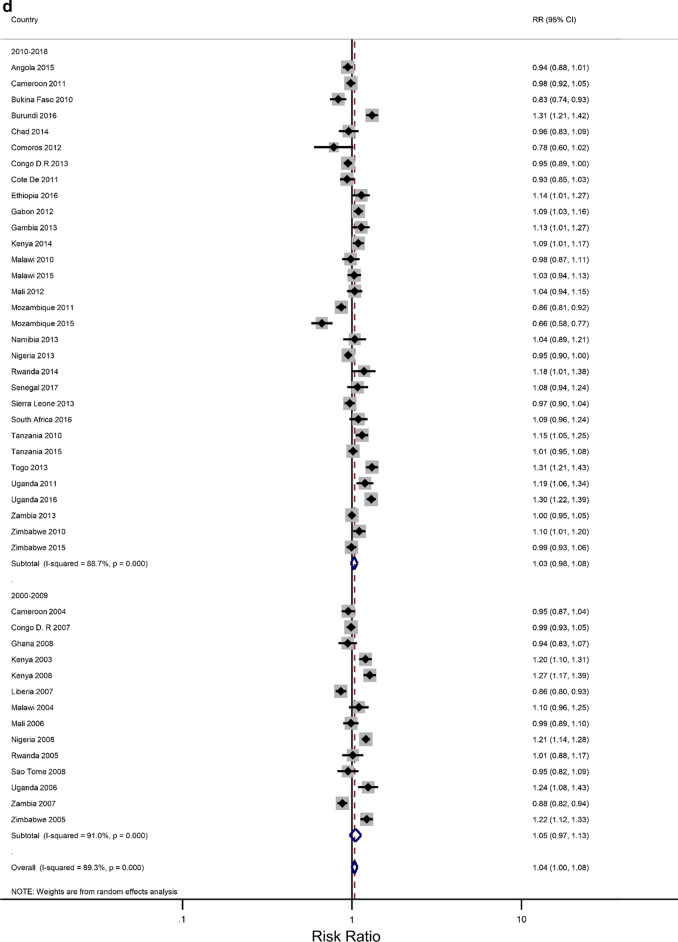

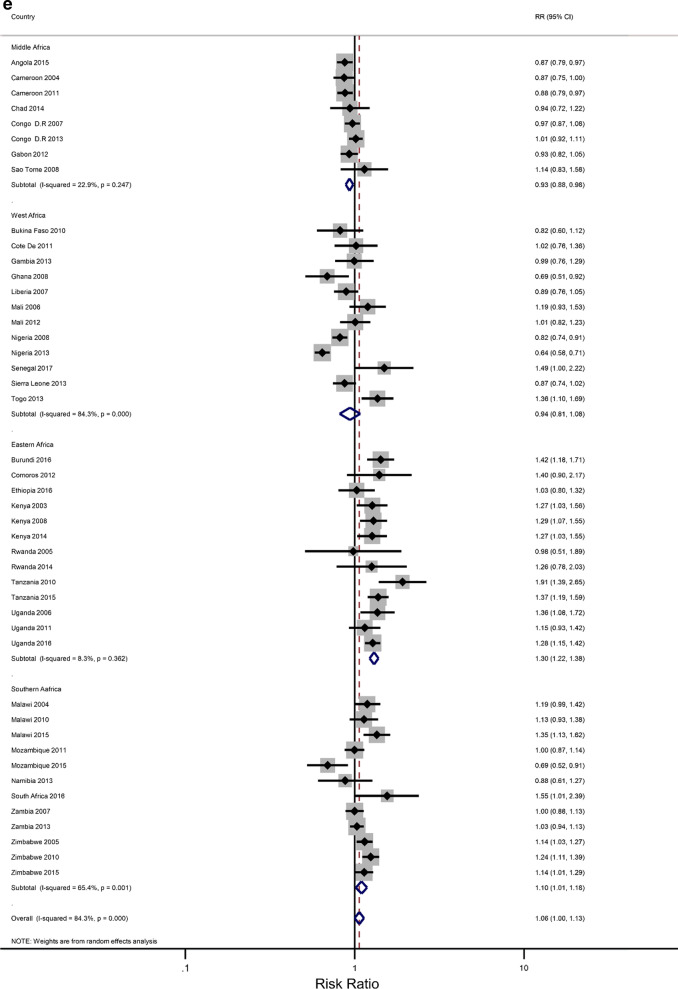

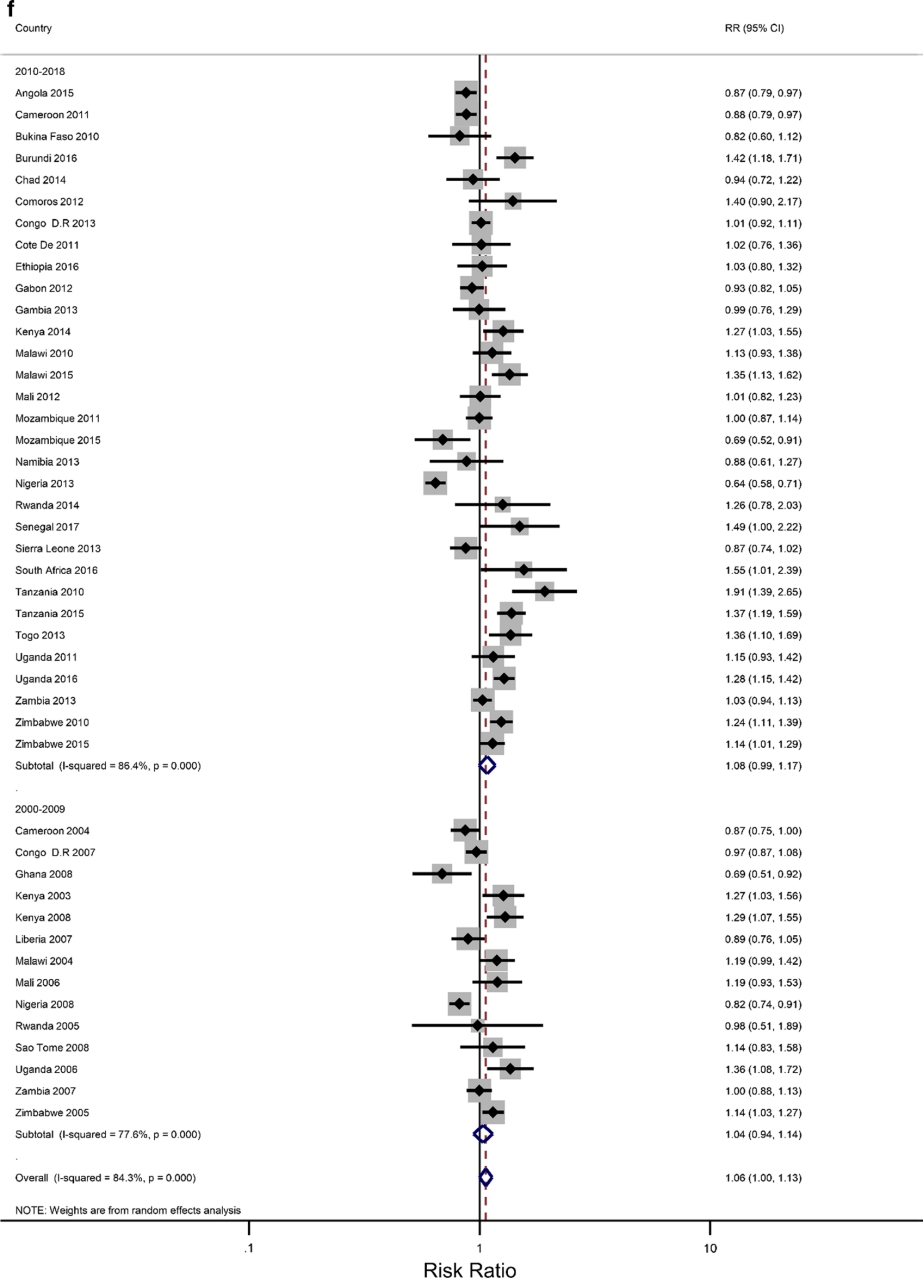

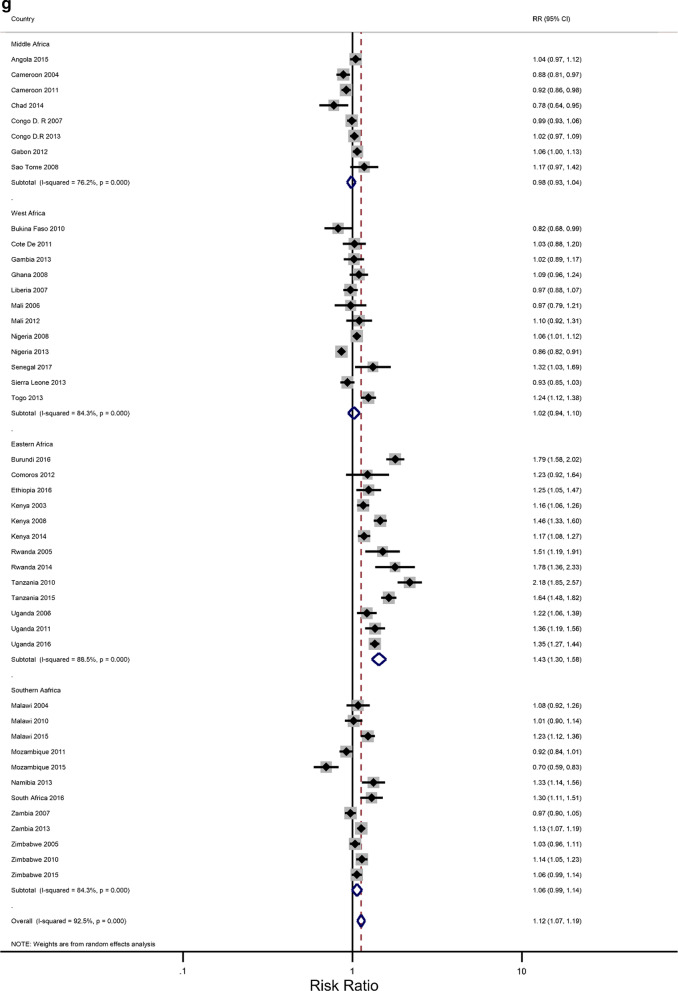

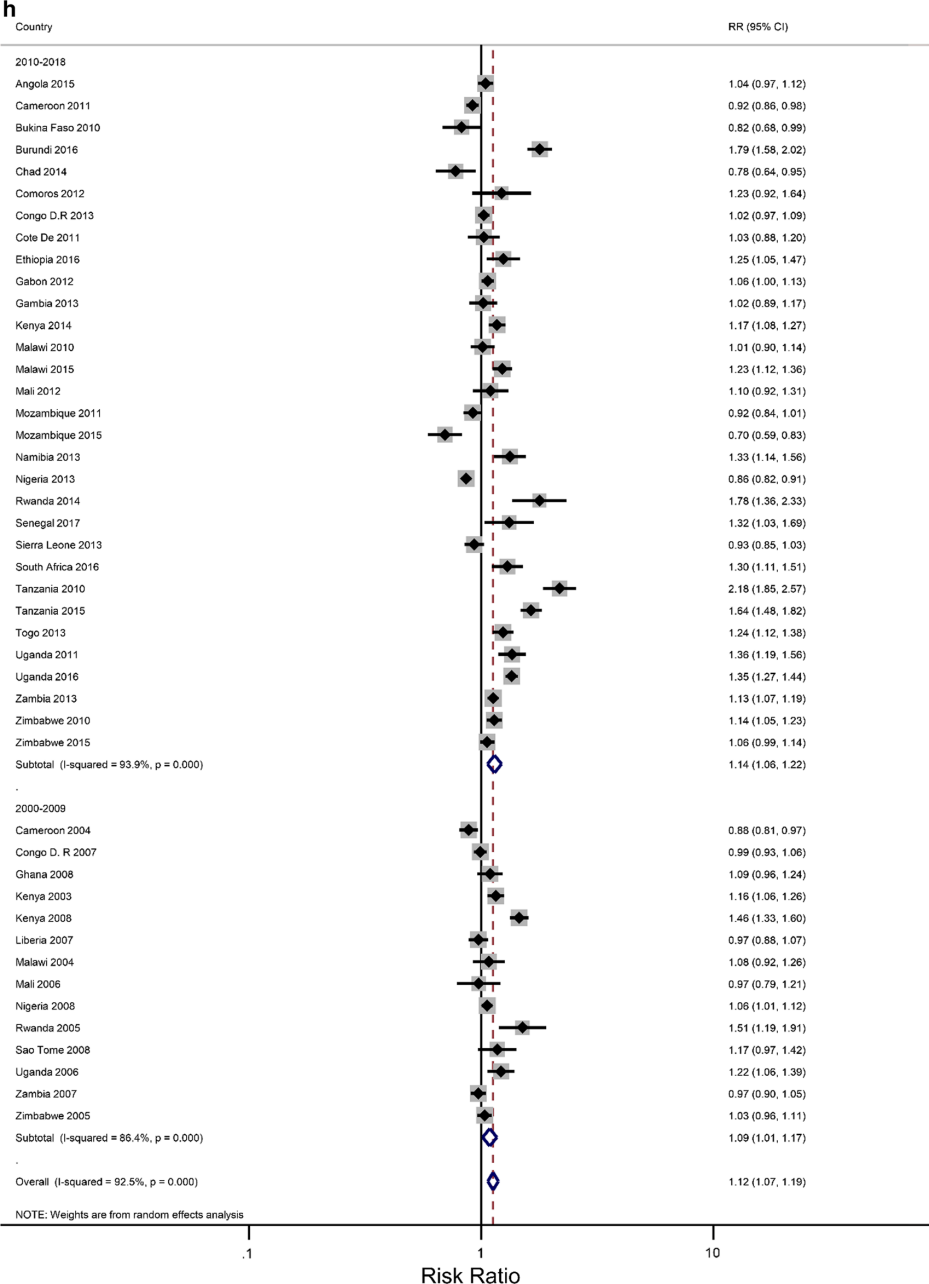
**a** The excess of Intimate Partner Violence prevalence among women aged 15–24 years in rural areas, compared to women of the same age group in urban areas, by region of Africa. The dotted vertical line represents the pooled risk ratio, with its 95% confidence interval. The solid vertical line at the value of 1 represents no difference in IPV rates between the types of residence. **b** The excess of Intimate Partner Violence prevalence among women aged 15–24 years in rural areas, compared to women of the same age group in urban areas, by period. The broken vertical line represents the pooled risk ratio, with its 95% confidence interval. The solid vertical line at the value of 1 represents no difference in IPV rates between the types of residence. **c** The excess of overall Intimate Partner Violence prevalence among women aged 25–49 years in rural areas, compared to women of the same age group in urban areas, by region of Africa. The broken vertical line represents the pooled risk ratio, with its 95% confidence interval. The solid vertical line at the value of 1 represents no difference in IPV rates between the types of residence. **d** The excess of Intimate Partner Violence prevalence among women aged 25–49 years in rural areas, compared to women of the same age group in urban areas, by period. The broken vertical line represents the pooled risk ratio, with its 95% confidence interval. The solid vertical line at the value of 1 represents no difference in IPV rates between the types of residence. **e** The excess of Intimate Partner Violence prevalence in women aged 15–24 with less than a secondary education, compared to women with at least a secondary school education, by region of Africa. The broken vertical line represents the pooled risk ratio, with its 95% confidence interval. The solid vertical line at the value of 1 represents no difference in IPV rates between the education levels. **f** The excess of Intimate Partner Violence prevalence in women aged 15–24 with less than a secondary education, compared to women with at least a secondary school education, by region of Africa. The broken vertical line represents the pooled risk ratio, with its 95% confidence interval. The solid vertical line at the value of 1 represents no difference in IPV rates between the education levels. **g** The excess of Intimate Partner Violence prevalence in women aged 25–49 with less than a secondary education, compared to women with at least a secondary school education, by region of Africa. The broken vertical line represents the pooled risk ratio, with its 95% confidence interval. The solid vertical line at the value of 1 represents no difference in IPV rates between the education levels. **h** The excess of Intimate Partner Violence prevalence in women aged 25–49 with less than a secondary education, compared to women with at least a secondary school education, by period. The broken vertical line represents the pooled risk ratio, with its 95% confidence interval. The solid vertical line at the value of 1 represents no difference in IPV rates between the education levels

Figures 3a–h show estimated pooled associations between IPV in rural–urban residence or women educational attainment for each age group. Among the younger women, the overall IPV prevalence amongst rural areas was not significantly greater than for urban communities (RR = 0.97; 0.93–1.01), but the association was significant in the Eastern Africa region (RR = 1.05; 1.00–1.11). On the other hand, older women experienced significantly higher levels of IPV in rural areas than in urban areas (RR = 1.04; 1.00–1.08), and the association attenuated in the Eastern region ((RR = 1.16; 1.09–1.23). The pooled IPV among the less educated young women was significantly greater (RR = 1.06; 1.00–1.13) with, again, the Eastern region having a larger association (1.30; 1.22–1.38). In older women, the association between experiencing IPV and lower levels of education was statistically significant (RR = 1.07; 1.07–1.19). The effect size was greatly attenuated in the Eastern African region (RR = 1.43; 1.30–1.58).

## Discussion

The study has synthesized rural–urban residence and educational level differences in the prevalence of intimate partner violence against women aged 15–49 years in sub-Saharan Africa (SSA). Summary data from forty-four Demographic and Health Survey (DHS) datasets in 29 countries with a total of 233,585 women were analyzed. The overall prevalence of experiencing any form of IPV was 41.3% (95% CI 37.4–45.2). The most common forms of IPV reported included physical (31.5%: 27.9–35.1) and emotional (27.4%; 24.6- 30.2), followed by sexual (12.9%; 11.0–14.8). These findings mirror the pooled estimates of IPV found in [21, 43, 45, 47–49]. They confirm that the prevalence of all types of IPV, namely physical, emotional violence, and sexual are consistently higher in SSA compared to the other regions in the world [45]. This is despite previous studies using a different number of countries, regions, sources of data, and age bands of the women. The higher rates of intimate partner violence among women in Sub-Saharan Africa (SSA) are sustained by strong and rigid harmful gender norms that tolerate partner abuse and acceptance of wife-beating, high levels of poverty, and conflict areas [1–3, 10, 50].

The sub-region analyses revealed that the highest rates of IPV were experienced by women in Middle Africa (driven by Cameron, Gabon, and the Democratic Republic of Congo) followed by Eastern Africa (driven by Burundi, Kenya, and Uganda). Our findings are consistent with other studies conducted in SSA countries that found a higher prevalence of IPV in these regions compared to the other two regions. [21, 51, 52]. A possible explanation for this is the uncontrolled violation of human rights and partner in conflict and post-conflict countries of the Burundi, DRC, Uganda, and northern parts of Cameroon) [53, 54]. Another explanation is that the two regions have strong community gender norms that socially, economically, and educationally disempower women and where wife beating is accepted [1–3, 43, 48]. One more possible explanation for the higher prevalence of IPV, especially sexual violence, in Middle and Eastern Africa could be the submissiveness of women, leading to them being unlikely to perceive IPV as an act of abuse [21, 51, 52].

Our findings revealed that women living in rural areas were more likely to have experienced higher rates of IPV compared to women residing in urban areas, but the association was only significant in the Eastern African region. This is consistent with the findings in [19] that showed a higher prevalence of IPV, as well as a more accepting attitude toward various types of IPV such as wife-beating in rural areas compared to urban areas [18, 19, 43]. This could be because traditional gender norms are more prevalent in rural areas [55]. However, we found the IPV prevalence to be similar between rural and urban women in most countries. A possible explanation for this is that in rural areas, cultural beliefs and traditions such as wife inheritance, polygamy, and religious factors may complicate the disclosure of any experience of IPV [56].

The study also found that women with higher education were less likely to experience IPV compared to women with less education, which is consistent with the findings in [11–13, 16, 17, 57]. For, instance, a survey carried out in Uganda reported the same trend [58] indicating that attaining at least a secondary education level was protective against IPV [28]. Also, a study carried out by Kishor & Johnson [42] using DHS data documented that women with no education were more likely to report spousal violence compared to women with a primary, a secondary, or higher level of education in most countries globally. More so, the same was found in a study carried out in a developed country (United States) that found non-high school graduates were more likely to experience IPV compared to high school graduates [59]. A possible explanation for this is that educated women are more likely to be employed and empowered compared to the less educated women [42]. Another possible explanation is that women with higher levels of education will have more access to resources and are more likely to be empowered in their relationships. However, inconsistencies in the association of educational attainment and IPV have been found in some settings, especially where the woman has a higher education than her partner. For example, in Malawi, a study found that sexual IPV was more prevalent among women with a higher level of education [55]. This could have been that increased educational attainment is perceived as a threat to men’s status as head of the household or an artifact of the data as there were very small numbers of women in the higher education group. A study in Ethiopia found higher levels of IPV among women residing in rural areas regardless of the levels of their education, and it became more prevalent when the women were married to less educated husbands [4]. Despite these mixed associations between women’s education and their experience of IPV, higher levels of education are associated with increased egalitarian and just social system views, which are conducive to lower levels of controlling behaviors, thus a reduction in IPV [50].

The study also revealed that IPV prevalence estimates in the second period 2010–2018 were lower compared to the first 2000–2009. A possible explanation for this is the increased number of preventative measures aimed at empowering women economically and socially. Cash transfers have broadened women’s access to economic opportunities, which has resulted in improved intimate partner violence [60–62]. Interventions that involve families and the promotion of sexual health, gender power, and psychological well-being have been associated with reduced risk of IPV [63]. Another possible explanation is that progressive legislation and judicial systems such as arrests and a civil protection order may have deterrent effects on subsequent IPV perpetration and would-be IPV perpetrators [63, 64]. However, the benefits of the arrest or court interventions reducing intimate partner violence are limited and mixed, depending on socio-demographic characteristics of the individual victims and perpetrators, and community attitudes towards women roles [64]. Yet another possible explanation is that increased education and employment opportunities, particularly for women, in the region has led to a decline in strong gender attitudes such as legitimizing wife-beating [43, 65].

### Strength and limitations of the study and future research

The strength of this study is that the DHS surveys are nationally representative population-based surveys, leading to estimates that are generalizable at the population level as opposed to smaller studies that may be based on very specific subsets of the population. Also, the sampling methods and the instruments used adhere to the accepted ethical standards recommended for research on IPV [40, 41]. Furthermore, rather than providing an appraisal and a summary of IPV prevalence data as in [41, 46, 47], our study has synthesized evidence of the association of IPV against women and rural–urban residence and literacy, some of the two major risk factors of spousal violence against women in sub-Saharan Africa using readily and publicly available observational data. Thus, our study has provided findings that have showed which groups of women are at higher risk of IPV, which are  necessary for optimal IPV interventions.

However, the findings reported here could be subject to limitations because we carried out a secondary analysis of data that was already collected in each country. We did not have any control regarding data collection and data management procedures yet these data have been found to have constructs that may be operationally defined by a single survey item or a subset of test items that can lead to reliability and validity issues [66]. However, since all DHS surveys adhere to pre-specified and consistent guidelines, we expect very minimal differences in the data collections between the included surveys. Furthermore, the DHS lacks details in the nature and context of IPV, yet experiences may differ qualitatively across life stages and geographical contexts. Also, since we are relying on self-report of IPV, we do acknowledge that in certain countries there may be an underreporting of IPV due to the sensitive nature of the module. Several countries have not implemented the IPV module into their DHS, so these countries did not contribute to IPV data.

We acknowledge that we could have stratified our analyses on other known IPV confounders, such as poverty (as provided by the household wealth index); polygamy, alcohol abuse, spousal literacy [4, 45, 46]. In particular, excessive alcohol consumption by one or both partners, could lead to aggressive and intimidating behaviors and forced sexual activity, and other forms of controlling behavior and significant hardships, including financial difficulties [57, 67]. In this study, we have controlled for the impact of the SSA region, period, and woman’s age on the association between IPV and residence type and education attainment. Only the region influenced the associations found. On the role of poverty and its association with IPV, conflicting evidence has been found, either for women's experience of IPV or their partner’s perpetration of IPV. It has generally been shown that higher income is associated with several mediating pathways that are linked to a reduction in IPV among women such as food security and fewer arguments over the lack of resources in the household [50, 61]. Women in households that are below the poverty line tend to feel insecure, which leads to submissive behaviors and prolonged abusive relationships. On the other hand, while women’s experience of IPV increases with higher levels of food insecurity and lower-income [50], women who contribute more financially than their partners have reported increased IPV risks as was found in a Tanzanian study [61]. For men, being economically marginalized leaves them with a perceived loss of `masculinity’ and `respect' on which relationships are thought to be based. In their frustration and anger, such men resort to using violence and control over women to assert their dominance in relationships and regain their masculinity [50].

Our findings could be affected by the definition and levels of the exposure variables used in the analysis. The definition of rural–urban as used in DHS datasets varies from country to country, even within the sub-Saharan African region. There is never any definition of the rural–urban in the DHS surveys nor addressed in a DHS report. As the DHS surveys are conducted in conjunction with the country’s Ministry of Health (MoH) and National Statistical Office (NSO), the surveys always adopt the country's urban–rural definition. So, the urban–rural definition is never addressed in a DHS report. One may need to check the definition for a specific country from the country's statistical office or government website (e.g. as used in their population and housing censuses). The definition could be based on population size or infrastructure. For example, in Botswana, the definition of urban is “Agglomeration of 5 000 or more inhabitants where 75% of the economic activity is non-agricultural.”, whereas in Malawi urban is defined as “All townships and town planning areas and all-district centers.” [68]. Similarly, education data in DHS varies from country to country. Years of education completed and education levels vary from country to country and may change over time within a country. Thus, respondents reporting the highest level of schooling attended and the highest grade completed at that level are country-specific [69].

We are aware that we could have opted for individual patient data (IPD) analysis with a multi-level approach that accommodates country-level. However, we opted for a meta-analysis of summary IPV statistics as we believe aggregated reporting at a country level of a critical public health issue is essential to identify vulnerable groups. In this way, our analysis has provided countries and relevant stakeholders with pooled IPV data for use in measuring progress towards the 2030 women's health goals, especially in low-income and middle-income countries (LMICs) [45]. Moreover, several studies have shown that both approaches of meta-analyses yield highly comparable results [70–72]. In particular, a recent study has shown that theoretically and numerically a summary-statistics-based meta analysis is asymptotically efficient as individual patient data analysis when the random effects (in both models) follow Gaussian distribution and estimation of summary statistics was done by maximum likelihood [73]. In this study, we performed Gaussian random-effects meta-analyses on IPV summary statistics that were obtained by maximum likelihood estimation. In hindsight, country-level summaries used for comparisons and synthesis in our study could have masked intra-country variations in IPV, which could be large as shown in a meta-analysis of IPV in Ethiopia [74].

## Conclusion

The study has found that the prevalence of IPV against women was higher among women who had lower education levels and resided in rural areas. Interventions that improve access to increased formal education among women, especially in the rural areas would help in reducing all forms of violence among girls and women across the sub-Saharan African region. Further such interventions should be structured in such a way that regional contexts, beliefs, and customs regarding women’s roles, rights, and decision-making in society are taken into account.

## Supplementary Information

**Additional file 1:** **Figure A1**: Prevalence of any IPV among women aged 15–49 years according to women’s residence type in each country and year. **Figure A2**: Prevalence of any IPV among women aged 15–49 years according to women’s level of education in each country and year.

**Additional file 2:** **Figure B1**: Prevalence of any IPV among women aged 15–24 years in each country and year by region of Africa. **Figure B2**: Prevalence of any IPV among women aged 25–49 years in each country and year by region of Africa. **Figure B3**: Prevalence of any IPV among women aged 15–24 years in each country and year by the period of DHS survey. **Figure B4**: Prevalence of any IPV among women aged 25–49 years in each country and year by the period of DHS survey.

## Data Availability

The datasets used in this study are available from the DHS website https://dhsprogram.com/Data/ upon request from the MEASURE DHS program team.

## Abbreviations

CI: Confidence interval
CTS: Conflict Tactics Scale
DV: Domestic violence
DRC: Democratic Republic of Congo
DHS: Demographic Health Survey
LIMIC: Low Income and Middle-Income Countries
IPD: Individual patient data
SDG: Sustainable Development Goals
IPV: Intimate partner violence
SSA: Sub Saharan Africa
PSU: Primary sampling unit
RR: Relative risk
USAID: United States Agency for International Development
MEASURE: Monitoring and Evaluation to Assess and Use Results
