# Supplementary material for: Effects of rural–urban residence and education on intimate partner violence among women in Sub-Saharan Africa: a meta-analysis of health survey data
Source: BMC Womens Health. 2021 Apr 13;21:149. doi: 10.1186/s12905-021-01286-5 (PMC8045348; doi:10.1186/s12905-021-01286-5)
Supplement: Supplementary file 1 — Additional file 1: Figure A1: Prevalence of any IPV among women aged 15–49 years according to women’s residence type in each country and year. Figure A2: Prevalence of any IPV among women aged 15–49 years according to women’s level of education in each country and year. [file 12905_2021_1286_MOESM1_ESM.docx]

Figure A1:

Figure A2:
