# Supplementary material for: Effects of rural–urban residence and education on intimate partner violence among women in Sub-Saharan Africa: a meta-analysis of health survey data
Source: BMC Womens Health. 2021 Apr 13;21:149. doi: 10.1186/s12905-021-01286-5 (PMC8045348; doi:10.1186/s12905-021-01286-5)
Supplement: Supplementary file 2 — Additional file 2: Figure B1: Prevalence of any IPV among women aged 15–24 years in each country and year by region of Africa. Figure B2: Prevalence of any IPV among women aged 25–49 years in each country and year by region of Africa. Figure B3: Prevalence of any IPV among women aged 15–24 years in each country and year by the period of DHS survey. Figure B4: Prevalence of any IPV among women aged 25–49 years in each country and year by the period of DHS survey. [file 12905_2021_1286_MOESM2_ESM.docx]

Figure B1: Prevalence of any IPV among women aged 15-24 years in each country and year by region of Africa.

Figure B2: Prevalence of any IPV among women aged 25-49 years in each country and year by region of Africa

Figure B3: Prevalence of any IPV among women aged 15-24 years in each country and year by period of DHS survey.

Figure B4: Prevalence of any IPV among women aged 25-49 years in each country and year by period of DHS survey
